# A Rim-Enhanced Mass with Central Cystic Changes on MR Imaging: How to Distinguish Breast Cancer from Inflammatory Breast Diseases?

**DOI:** 10.1371/journal.pone.0090355

**Published:** 2014-03-05

**Authors:** Lijun Wang, Dengbin Wang, Xiaochun Fei, Mei Ruan, Weimin Chai, Lin Xu, Xiaoxiao Li

**Affiliations:** 1 Department of Radiology, Xinhua Hospital, Shanghai Jiao Tong University School of Medicine, Shanghai, China; 2 Department of Pathology, Ruijin Hospital, Shanghai Jiao Tong University School of Medicine, Shanghai, China; 3 Department of Radiology, Ruijin Hospital, Shanghai Jiao Tong University School of Medicine, Shanghai, China; West German Cancer Center, Germany

## Abstract

**Objective:**

To evaluate the capacity of magnetic resonance imaging (MRI) to distinguish breast cancer from inflammatory breast diseases manifesting as a rim-enhanced mass with central cystic changes.

**Materials and Methods:**

Forty cases of breast cancer and 52 of inflammatory breast diseases showing a rim-enhanced mass with central cystic changes were retrospectively reviewed. All cases underwent dynamic contrast-enhanced MRI and 31 of them underwent diffusion-weighted imaging (DWI). Morphological features, dynamic parameters and apparent diffusion coefficient (ADC) values were comparatively analyzed using univariate analysis and binary logistic regression analysis.

**Results:**

Breast cancer had a significantly thicker wall than the inflammatory breast diseases (P<0.001) while internal enhancing septa were more common in inflammatory breast diseases (P = 0.003). On DWI, 86.7% of breast cancers demonstrate a peripheral hyperintensity whereas 93.8% of inflammatory breast diseases had a central hyperintensity (P<0.001). Compared to the inflammatory breast diseases, breast cancers had a lower ADC value for the wall (1.09×10^−3^ mm^2^/s vs 1.42×10^−3^ mm^2^/s, P<0.001) and a higher ADC value for the central part (1.94×10^−3^ mm^2^/s vs 1.05×10^−3^ mm^2^/s, P<0.001).

**Conclusions:**

Both breast cancer and inflammatory breast diseases could present as a rim-enhanced mass with central cystic changes on MRI. Integrated analysis of the MR findings can allow for an accurate differential diagnosis.

## Introduction

Rim enhancement is defined as more pronounced at a mass' periphery on dynamic contrast-enhanced magnetic resonance imaging (DCE-MRI) [1]. There can be fibrosis or necrosis in the central area. A rim-enhanced mass with central fibrosis usually shows delayed centripetal progression, however, there is no enhancement in the necrotic area. A rim-enhanced mass with central cystic changes usually shows central high signal intensity (SI) on T2-weighted imaging (T2WI) and has been observed in a broad spectrum of benign and malignant breast diseases. In benign diseases, it is most commonly seen in inflammatory breast diseases such as different types of mastitis with abscess formation, inflammatory cysts or galactoceles, and fat necrosis [2]–[6]. However, it also could be seen in malignant tumors like invasive ductal carcinoma (IDC), intracystic papilloma or papillary carcinoma, and some rare breast cancers like metaplastic cancer [2], [7]–[10]. Solid nodular protrusion in the wall-like peripheral part of the cystic mass has been described as a specific sign of intracystic papilloma or papillary carcinoma [10]. However, breast cancers like IDC and metaplastic cancer with central necrosis are difficult to be differentiated from the inflammatory breast diseases, especially when there is prominent necrosis or inflammatory changes in the skin. The overlapping imaging features make an accurate diagnosis difficult.

Both breast cancer and inflammatory breast diseases could have false negative findings on mammography and ultrasonography and it is hard to differentiate these two kinds of diseases using mammography and ultrasonography [3], [11]. Data from DCE-MRI are of limited value for differentiating breast cancer from inflammatory breast diseases [4], [11], [12]. Recently, T2WI has been considered as a tool to differentiate the two conditions [3], [11]–[13]. In the brain, diffusion-weighted imaging (DWI) has been shown to be effective to distinguish a cystic tumor from an abscess [14]. However, in the breast, for cancers and inflammatory diseases showing a rim-enhanced mass, DWI has only been reported in a few articles [15]–[17].

Therefore, the purpose of this study was to retrospectively evaluate the capacity of MRI to distinguish breast cancers from the inflammatory breast diseases manifesting as a rim-enhanced mass with central cystic changes.

## Materials and Methods

### Patient selection

This clinical research complies with the declaration of Helsinki. Our study was approved by the institutional review board of Xinhua Hosptial. The data were analyzed anonymously and we confirmed that there was no private information in the manuscript and the images. Therefore, the institutional review board waived the need for written informed consent from the participants for this retrospective study. This study evaluated all the patients admitted to our hospital between September 2009 and June 2013 with breast cancers or inflammatory breast diseases which showed a rim-enhanced mass with central cystic changes on DCE-MRI. Patients who had undergone biopsy before MRI were excluded. The cases of introcystic papilloma or papillary carcinoma were also excluded because there were distinct findings for these two diseases. Forty cases of breast cancer and 52 cases of inflammatory breast diseases were enrolled. There was one male in the inflammatory breast diseases group. In all patients, diagnosis was confirmed by core needle biopsy (CNB, n = 13) or by surgical pathology (n = 79). The 40 cases of breast cancer included 34 invasive ductal carcinomas of no special type, 2 invasive ductal carcinomas with neuroendocrine differentiation, 2 metaplastic carcinomas, 1 mixed lobular carcinoma, and 1 neuroendocrine carcinoma. Two cases had a history of previously contralateral breast cancer. Among the 40 cases, 24 (60.0%) were triple-negative breast cancer (TNBC), 7 (17.5%) were locally advanced breast cancers (LABC) and 2 (5.0%) were inflammatory breast carcinomas (IBC). The 52 benign cases included 20 cases of simple mastitis with abscess formation, 9 inflammatory cysts, 10 inflamed dilated ducts with cyst formation, 7 granulomatous mastitides without further classification, 2 duct ectasias, 1 fat necrosis, 1 lactating abscess, 1 inflammatory galactocele and 1 tuberculous mastitis. One female in the inflammatory breast diseases group was lactating. All the patients complained of a palpable breast mass. Inflammatory symptoms (pain, erythema or heat) were recorded. There were two patients with a high white blood cell (WBC) counting in the inflammatory breast diseases group and no patient in the breast cancer group had a high level of WBC (P = 0.503).

### Imaging techniques

MRI was performed with two different scanners, a 1.5-T dedicated spiral breast MRI system with a single channel quadrature breast coil (Aurora Systems, USA), and a 3.0-T whole body MRI scanner with a eight-channel phase-array breast coil (Signa HDxt, GE Medical System). All patients were placed in the prone position, feet first, on the table inside the MRI scanner. For the dynamic imaging, the gadolinium-diethylenetriamine pentaacetic acid (Gd-DTPA; Magnevist; Bayer-Schering Pharma AG, Berlin, Germany) was intravenously administered at a dose of 0.2 mmol per kilogram of body weight as a bolus at a flow rate of 2 ml/s followed by a 20-mL normal-saline flush.

MR imaging of 42 patients were performed using a 1.5-T MRI system, including 15 patients in the breast cancer group and 27 in the inflammatory breast diseases group. The spiral axial screen mode was used. Dynamic imaging of both breasts was obtained before and at 180 s, 360 s, and 540 s after the injection of Gd-DTPA. The following parameters were used for this sequence: repetition time (TR), 4.8 ms; echo time (TE), 29 ms; thickness, 1.125 mm; FOV, 36 cm; matrix, 360×360×128. MR imaging of the other 50 patients were performed using a 3.0-T whole body MRI scanner, including 25 patients in the breast cancer group and 25 in the inflammatory breast diseases group. After a localizer on the three orthogonal planes and coil calibration was taken, axial short tau inversion recovery (STIR) was obtained with the following parameters: TR, 7060 ms; TE, 35.2 ms; inversion time (TI), 170 ms; time of acquisition (TA), 120 s; thickness, 4.0 mm; FOV, 32 cm; and matrix, 320×192. Then, DWI with b-values of 0 and 600 s/mm^2^ (n = 6) or 800 s/mm^2^ (n = 25) were applied in the axial plane. The other parameters for DWI were, for 600 s/mm^2^: TR, 5950 ms; TE, 64.6 ms; thickness, 4.0 mm; FOV, 32 cm; and matrix, 160×160. For 800 s/mm^2^, parameters were: TR, 5125 ms; TE, 66.4 ms; thickness, 4.0 mm; FOV, 32 cm; and matrix, 128×128. Dynamic imaging of both breasts with the Vibrant sequence was obtained before and at 54 s, 108 s, 162 s, 216 s, and 270 s after the injection of Gd-DTPA. The following parameters were used for this sequence: TR, 4.3 ms; TE, 2.1 ms; TI, 14 ms; thickness, 1.2 mm; FOV, 42 cm; matrix, 416×320.

### Image analysis

All the images in this study were reviewed retrospectively and all the post-processing was performed by two breast radiologists (3–4 years of practice) in consensus. The disputes were resolved via consultation of a third experienced breast radiologist (DW, 20 years of practice). The radiologists were unaware of the clinical information and histopathological diagnosis. The first post contrast sequence (180 s) for 1.5-T MRI and the second post contrast sequence (108 s) for 3.0-T MRI were referred to as early-enhanced images to evaluate the enhancement of a lesion. The morphology of each lesion was classified according to the Breast Imaging Reporting and Data System (BI-RADS) MRI lexicon developed by the American College of Radiology in 2003 [1]. For patients with multiple lesions, the imaging features of the biopsied one were taken for analysis.

Post-processing included subtraction of the pre-contrast images from the dynamic axial T1 sequence on a pixel-by-pixel basis; the time-signal intensity curve (TIC) was obtained in both scanners. A region of interest (ROI) for the TIC was drawn freehand on the wall of the mass and care was taken to avoid central cystic regions. When the wall of the mass was too thin to establish a ROI, we usually magnified the image and set the ROI inside the wall. Additionally, on the Aurora post-processing workstation, the TA of each sequence was 180 s while the center of the 3D k-space cube was filled at 90 s after initiation of acquisition. So the three time points for the three post contrast sequences on the TIC were 90 s, 270 s, and 450 s. The color mapping was also obtained. In the color maps, blue indicates no enhancement and represents cystic areas; yellow indicates a persistent pattern; pink indicates a plateau pattern; and red indicates a washout pattern. For the 3.0-T MRI system, the DWI and the apparent diffusion coefficient (ADC) value mapping were obtained on the GE post-processing workstation. We compared the imaging findings from DCE-MRI, STIR and the DWI to identify the wall and the central cystic area of the rim-enhanced mass. Three ROIs in different places were drawn freehand on the wall and the central cystic area on the ADC value mapping images, respectively, and the corresponding ADC values were then automatically obtained. Like with the TIC, the image was properly magnified to place the ROI when there was a thinner wall. The size of ROI ranged from 6–30 mm^2^. The three measurements were averaged and the mean was used as the ADC value.

Duration was defined as the time between when clinical symptoms were reported and the MRI examination. The adjacent vessel sign (AVS) was considered present if a vessel leading to a lesion could be clearly delineated on subtracted images [18]. Skin thickening was considered to be present if the skin is focally or diffusely thicker than normal [1]. Skin invasion was defined as abnormal enhancement within the skin, which is often also thickened [1]. On T2WI, prepectoral edema was considered present if the SI was similar to that of water, in the area in front of the pectoral muscles [13]. Axillary lymphadenopathy was concluded if thickened, eccentric, or irregular abnormal cortexes, or rounded lymph nodes with loss of fatty hila were present [19]. The retroareolar region was considered involved if the lesion localized or extended to the subareolar area.

### Statistical analysis

For comparisons of the categorical variables between the two groups, the Chi-squared and Fisher exact tests were employed. The Student t-test was used to analyze the differences in age and ADC values between the two groups. The Mann-Whitney U test was used to compare the duration, tumor size, and the thickness of the wall in the two groups. A binary, multivariate logistic regression analysis was performed with selected variables to identify clinical or imaging characteristics that differed between the two groups. Analyses were conducted with the SPSS 16.0 analysis software package, and a *P* value less than 0.05 was regarded as statistically significant.

## Results

### Baseline characteristics

The clinical characteristics for patients with breast cancer and inflammatory breast diseases were summarized in Table 1. The mean age of patients with breast cancer was 55.4±13.9 years and for those with inflammatory breast diseases, 44.5±11.3 years (P<0.001). No significant differences in duration, inflammatory symptoms, or family history of breast cancer were observed between the two groups.

**Table 1:** Clincal characteristics of breast cancer and inflammatory breast diseases.

Variables	Breast cancer	Inflammatory breast diseases	*P* Value
Age (years)^a^	55.4±13.9	44.5±11.3	<0.001
Duration (days)^b^	45(10–165)	30(7–90)	0.182
Inflammatory symptoms			
Pain	14(35.0%)	25(48.1%)	0.208
Erythema	2(5.0%)	7(13.5%)	0.317
Heat	1(2.5%)	6(11.5%)	0.221
Family history of breast cancer	1 (2.5%)	3(5.8%)	0.805
Total	40(100.0%)	52 (100.0%)	

a Data are expressed as mean ± standard deviation.

b Data are expressed as median with interquartile range in parentheses.

### DCE-MRI features

**Figure 1:**
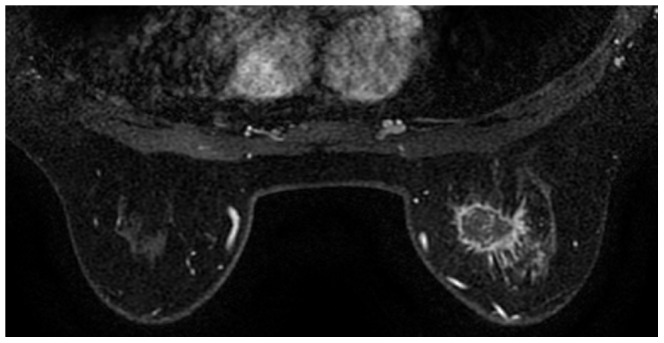
A 50 year-old woman in the breast cancer group. A painful mass was found in the right breast for 2 weeks. Pathology: invasive ductal carcinoma. T1-weighted contrast-enhanced MRI (108 s) reveals a rim-enhanced mass with a thin wall, an irregular shape and a spiculated margin.

**Figure 2:**
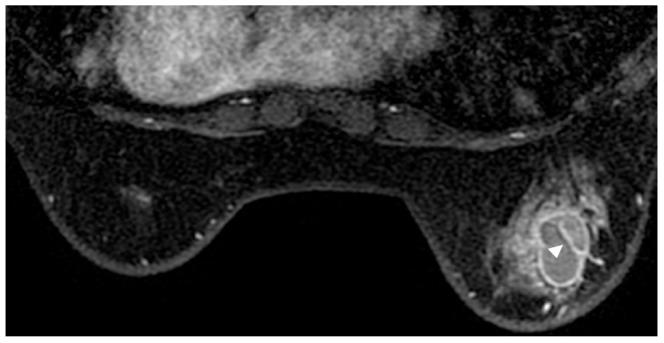
A 44 year-old woman in the inflammatory breast diseases group. A painful mass was found in the right breast for 3 days. Pathology: inflamed dilated ducts with cyst formation. T1-weighted contrast-enhanced MRI (108 s) reveals a rim-enhanced mass with a thin wall, an oval shape, internal septa (arrowhead), and a smooth margin.

MRI findings for patients with breast cancer and inflammatory breast diseases were summarized in Table 2. There was no significant difference between the two groups for the size of the lesion (P = 0.172). The thickness of the wall in breast cancer was significantly greater than that in inflammatory breast diseases (P<0.001). A round or oval shape was observed more often in the inflammatory breast diseases group (44.2%), while a lobulated or irregular shape was more common in the breast cancer group (80.0%, P = 0.015). Compared to inflammatory breast diseases, breast cancers had margins that were more commonly spiculated (25.0% vs 7.7%) (Figure 1), but were less frequently smooth (15.0% vs 17.3%) and irregular (60.0% vs 75.0%). However, these differences were not statistically significant (P = 0.072). Internal enhancing septa were more common in inflammatory breast diseases than breast cancer (50.0% vs 20.0%, P = 0.003) (Figure 2). Breast cancers more frequently showed a washout pattern of TIC and less frequently a persistent pattern than inflammatory breast diseases (P = 0.003).

**Table 2:** MRI findings of breast cancer and inflammatory breast diseases.

Variables	Breast cancer	Inflammatory breast diseases	*P* Value
Tumor size (mm)^a^	31.5(25.0–44.8)	27.5(19.3–44.0)	0.172
Thickness of the wall(mm)^a^	6.2(4.6–8.9)	3.5(2.4–5.7)	<0.001
Shape			0.015
Round or oval	8(20.0%)	23(44.2%)	
Lobulated or irregular	32(80.0%)	29(55.8%)	
Margin			0.072
Smooth	6(15.0%)	9(17.3%)	
Irregular	24(60.0%)	39(75.0%)	
Spiculated	10(25.0%)	4(7.7%)	
Internal enhancing septa			0.003
Yes	8(20.0%)	26(50.0%)	
No	32(80.0%)	26(50.0%)	
TIC			0.003
Persistent	7(17.5%)	20(38.5%)	
Plateau	2(5.0%)	10(19.2%)	
Washout	31(77.5%)	22(42.3%)	
The associated findings			
Retroareolar region involvement	15(37.5%)	27(51.9%)	0.169
Nipple inversion	13(32.5%)	13(25.0%)	0.428
AVS	39(97.5%)	41(78.8%)	0.008
Axillary lymphadenopathy	20(50.0%)	9(17.3%)	0.001
Skin thickening	15(37.5%)	14(26.9%)	0.279
Skin invasion	3(7.5%)	2(3.8%)	0.762
Prepectoral edema^b^	19(76.0%)	8(32.0%)	0.002
Total	40(100.0%)	52 (100.0%)	

TIC  =  time-signal intensity curve; AVS  =  the adjacent vessel sign.

a Data are expressed as median with interquartile range in parentheses.

b 25 cases of inflammatory breast diseases and 25 cases of breast cancers underwent axial short tau inversion recovery (STIR).

In addition, the breast cancer group more commonly showed AVS (P = 0.008), axillary lymphadenopathy (P = 0.001), and prepectoral edema (P = 0.002). No significant differences were found in the rates of retroareolar region involvement, nipple inversion, skin thickening, skin invasion between the two groups.

### DWI features

**Figure 3:**
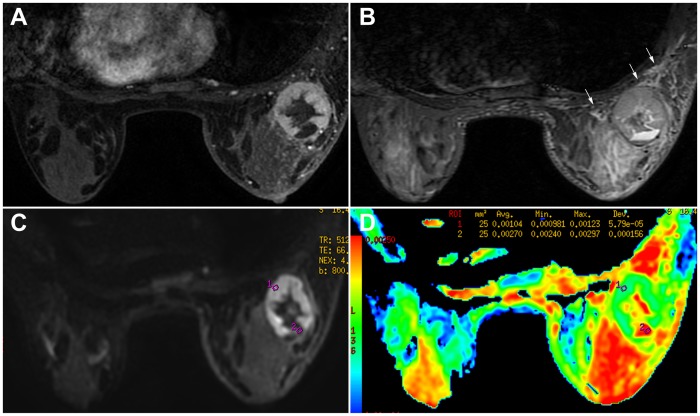
A 26 year-old woman in the breast cancer group. A painful mass was found in the right breast for 2 months. Pathology: invasive ductal carcinoma. **A**. T1-weighted contrast-enhanced MRI (108 s) reveals a rim-enhanced mass with a thick wall and smooth margin. **B**. STIR indicates the prepectoral edema (arrows) and partial of the non-enhancing central part corresponding to contrast-enhanced MRI presents as hyperintensity. **C**. The mass shows peripheral hyperintensity on DWI. **D**. The corresponding ADC map shows the ADC value of the wall is 1.04×10^−3^ mm^2^/s, the central part, 2.70×10^−3^ mm^2^/s.

**Figure 4:**
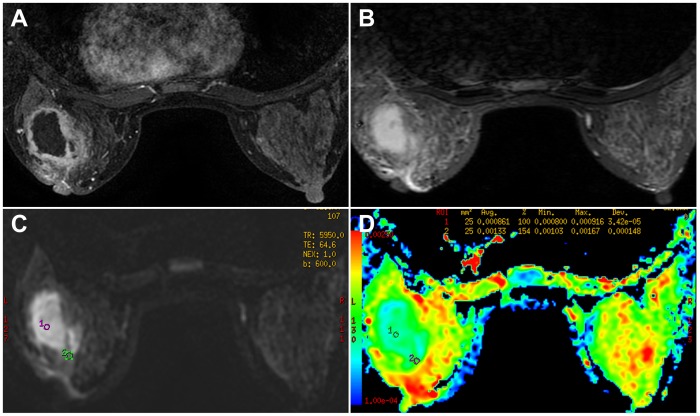
A 27 year-old woman in the inflammatory breast diseases group. A painful mass was found in the left breast for 1 month during breastfeeding. Pathology: lactating abscess. **A**. T1-weighted contrast-enhanced MRI (108 s) reveals a rim-enhanced mass with irregular margin. **B**. STIR indicates no prepectoral edema and the non-enhancing central part corresponding to contrast-enhanced MRI presents as hyperintensity. **C**. The mass shows central hyperintensity on DWI. **D**. The corresponding ADC map shows the ADC value of the wall is 1.33×10^−3^ mm^2^/s, the central part, 0.861×10^−3^ mm^2^/s.

DWI findings and the ADC values of the rim-enhanced mass in the two groups are summarized in Table 3. On DWI, 86.7% of breast cancers showed a peripheral hyperintensity (Figure 3) while 93.8% of the inflammatory breast diseases showed a central hyperintensity (Figure 4) (P<0.001). The mean ADC value in the wall of breast cancers and the inflammatory breast diseases were 1.09±0.18×10^−3^ mm^2^/s and 1.42±0.20×10^−3^ mm^2^/s, respectively, depicting a statistically significant difference (P<0.001). Similarly, the mean ADC value from the central part of breast cancers and the inflammatory breast diseases were 1.94±0.51×10^−3^ and 1.05±0.44×10^−3^ mm^2^/s, respectively, also indicating a statistically significant difference (P<0.001).

**Table 3:** DWI features and ADC values of the rim-enhanced mass between the two groups.

Variables	Breast cancer	Inflammatory breast diseases	*P* Value
Type			<0.001
Peripheral hyperintensity	13(86.7%)	1(6.2%)	
Central hyperintensity	2(13.3%)	15(93.8%)	
ADC values of the wall (×10^−3^ mm^2^/s)^a^	1.09±0.18	1.42±0.20	<0.001
ADC values of the central part (×10^−3^ mm^2^/s)^a^	1.94±0.51	1.05±0.44	<0.001
Total	15(100.0%)	16 (100.0%)	

DWI  =  diffusion-weighted imaging; ADC  =  apparent diffusion coefficient.

a Data are expressed as mean ± standard deviation.

### Logistic analysis

In a multivariate logistic regression model, we examined the following factors: age, thickness of the wall, shape, internal enhancing septa, TIC, AVS, and axillary lymphadenopathy (Table 4). This analysis demonstrated that older age, thicker wall, and axial lymphadenopathy were independent risk predictors of breast cancer, and that internal enhancing septa was an independent risk predictor of inflammatory breast diseases.

**Table 4:** Multivariate analysis of age, thickness of the wall, shape, internal enhancing septa, TIC, AVS, and axillary lymphadenopathy between the two groups.

Variables	Odds-ratio	95% CI	*p* Value
Age	1.11	1.05–1.18	<0.001
Thickness of the wall	1.40	1.11–1.76	0.004
Internal enhancing septa	0.16	0.04–0.62	0.008
Axillary lymphadenopathy	5.61	1.38–22.9	0.016

CI  =  confidence interval.

## Discussion

Both breast cancer and inflammatory breast diseases could show various enhancement patterns on DCE-MRI. A rim-enhanced mass with central cystic changes is commonly seen in inflammatory breast diseases; however, it could be also seen in breast cancer. The differential diagnosis remains a challenge. Therefore, we performed this study to evaluate clinical and imaging results from MRI of breast cancers and inflammatory breast diseases, which showed a rim-enhanced mass with central cystic changes, and to identify useful features that can help differentiate these two conditions to a great extent.

The average age of breast cancer patients in our study was 55.4 years, older than the patients with inflammatory breast diseases, which is consistent with previous reports [7], [11]. In our study, inflammatory symptoms were considered present if a patient had any symptoms of pain, erythema or heat. The results revealed no significant difference between the two groups regarding inflammatory symptoms, which is consistent with the previous study [11]. Breast cancer demonstrated a thicker wall than the inflammatory breast diseases. However, when there was a large central acellular zone, the wall of breast cancer was very thin [7], which was similar to that of inflammatory disease. Most breast cancers had a lobulated or irregular shape, whereas most inflammatory breast diseases showed a round or oval shape.

Renz et al. [11] have found that non-mass-like lesions were detected in all the cases of acute mastitis and inflammatory breast carcinomas; non-mass-like enhancement was observed adjacent to the mass. In our study, margins of both the breast cancer and inflammatory disease were very commonly irregular, in part because of the non-mass-like enhancement lesion adjacent to the mass. The TNBC has been shown to be commonly higher-grade differentiation, smooth mass margins and rim enhancement [17]. In the present study, 15.0% of breast cancers showed a smooth margin, which may be related to the fact that most of the breast cancers included in our study were TNBC. Breast cancers showed a spiculated margin more frequently than inflammatory breast diseases, although this was not statistically significant. Inflammatory breast diseases showed more frequently internal enhancing septa than breast cancers. We postulate that this is because of inflammation and dilation of ducts in these conditions.

Many studies have shown that most cases of mastitis show a persistent or plateau pattern, which helps to differentiate the condition from malignant lesions [11], [12], [20]. In our study, breast cancer showed a washout pattern more frequently than inflammatory breast diseases. This finding is consistent with previous results [11]. In the inflammatory breast diseases group in this study, a persistent pattern (38.5%) was almost as frequent as a washout pattern (42.3%). Angiogenesis is an important process of both malignancy and inflammatory disease. Compared to breast cancer, chronic inflammation usually coincides with an increased formation of new blood vessels with complete and mature structures, with a normal blood supply [21]. Therefore, some inflammatory diseases show a persistent pattern. In contrast, the wall of abscesses had more new vessels and a higher permeability, and therefore this type of lesion could have a washout pattern. Because of angiogenesis, the AVS has been considered as an indicator of malignancy [18], [22]. Inflammatory breast diseases have been reported as the most common benign disease with positive AVS [18]. In this study, AVS commonly occurred in both inflammatory breast diseases (78.8%) and breast cancer (97.5%). Although there was a significant difference between the two groups, it would be difficult to use AVS to distinguish the conditions in clinical context.

Axillary lymphadenopathy implied lymph node metastasis in breast cancer group, while in inflammatory disease it indicates reactive lymph node swelling. It was reported that a tumor located in the retroareolar region had a higher rate of lymph node metastasis because the lymphatic vessels converge in the retroareolar area [23]. In our study, although retroareolar region involvement was found in more frequently in the inflammatory breast diseases group (51.9%) than the breast cancer group (37.5%), axillary lymphadenopathy was more common in breast cancer (50.0%) than in inflammatory breast diseases (17.3%), which could be useful for differential diagnosis. We also examined other signs, such as nipple inversion, skin thickening, and skin invasion, which occurred in both groups without significant statistical difference. Skin invasion usually shows abnormal enhancement in the skin and related to tumoral invasion [12]. However, inflammatory diseases could also involve the skin and form abscess in the skin. Hence, skin invasion has a limited value to make a differential diagnosis.

Previous study have suggested that IBC and a tumor with a larger size usually show prepectoral edema, because of lymphatic invasion of cancer cells [13]. However, prepectoral edema occurs less commonly in mastitis, because edema of mastitis results from inflammation of infection instead of lymphatic invasion of cancer cells [11]–[13]. In our study, prepectoral edema was found more commonly in breast cancer (76.0%) than inflammatory breast diseases (32.0%). In the breast cancer group, there were 7cases of LABC and 2 cases of IBC, which may contribute to the higher rate of the prepectoral edema. However, the perifocal edema caused by the inflammation in an inflammatory lesion could be recognized as prepectoral edema when the lesion located close to the pectoral muscle. Therefore, a prospective study with a larger sample size is needed to evaluate the value of prepectoral edema for differential diagnosis.

DWI has been confirmed useful in differentiating the causes of ring-enhancing brain lesions [14]. Abscesses show diffusion restriction of the central part due to purulent material in the cavity. However, neoplastic lesions with central cystic changes did not show central diffusion restriction, due to less viscous and cellular materials in the cavity [14]. This difference was also found in breast lesions. Orguc et al. [16] have reported that, on DWI, a tuberculous abscess was manifested as a central hyperintensity, while a malignant tumor with central necrosis was revealed as a peripheral hyperintensity. In our study, 86.7% of breast cancers showed peripheral hyperintensity and a lower ADC value of the wall; however, 93.8% of inflammatory breast diseases showed a central hyperintensity and a lower ADC value of the central part.

Previous pathologic findings have indicated that there can be inflamed dilated ducts, inflammatory cysts, and abscesses in inflammatory breast diseases [3]. Therefore, we hypothesize that, as to the inflammatory breast diseases, inflammatory cells and debris inside the cysts, dilated ducts and abscesses likely contribute to the central hyperintensity on DWI. In contrast, as to breast cancer, the high cellularity of tumor cells in the wall likely contributes to the peripheral hyperintensity on DWI. The differences in DWI findings and ADC values between breast cancer and inflammatory breast diseases could help differentiate these conditions for diagnosis.

On DWI, there was one case of inflammatory disease with a peripheral hyperintensity and two cases of breast cancers with a central hyperintensity. Possible explanations for a breast abscess showing a peripheral hyperintensity could be the variable viscosity of an abscess, the age of an abscess, or a partially treated cavity [14]. Squamous cell carcinoma could also occur with a rim-enhanced mass containing inflammatory debris and tumor cells in the cavity [9], which could also show a central hyperintensity. Under these conditions, a biopsy is required to exclude the malignancy.

The main limitation of the present study is the small sample size. Only a small number of cases underwent DWI and T2WI,so we didn't put the prepectoral edema, DWI findings, and ADC values in the logistic regression analysis because of missing values. Another limitation is that there were two scanners with different magnetic sequences and for 3.0-T MRI, two different b values were used. Although the morphological features and kinetic curves were little influenced [3], [17], the difference in ADC values between different b values needed to be considered. A prospective MRI study with a larger sample size and a single MR imaging system is needed to verify the utility of DWI and T2WI for differentiating inflammatory breast diseases from cystic neoplasm.

In conclusion, both breast cancer and inflammatory breast diseases could present as a rim-enhanced mass with central cystic changes on MRI. Compared with inflammatory disease, breast cancer usually has a higher age of onset, a thicker wall, and with DWI, shows a peripheral hyperintensity with a lower ADC value of the wall. In contrast, inflammatory breast diseases more frequently show internal enhancing septa and with DWI, a central hyperintensity, and a lower ADC value of central part. The combination of multiple MRI features may thus provide the valuable information for differential diagnosis.
